# Making post-exposure prophylaxis effective for leprosy elimination: Insights from a multi-country study on low-endemic settings

**DOI:** 10.1371/journal.pntd.0013716

**Published:** 2025-11-18

**Authors:** Stephanie M. Tess van der Putten Hadik, Anil Fastenau, Anne Schoenmakers, Nimer Ortuño-Gutiérrez, Ricky Janssen

**Affiliations:** 1 School of Medicine, Dentistry and Nursing, University of Glasgow, Glasgow, United Kingdom; 2 Department of Health, Ethics & Society, Maastricht University, Maastricht, the Netherlands; 3 German Leprosy and Tuberculosis Relief Association (DAHW), Wuerzburg, Germany; 4 Marie Adelaide Leprosy Center, Karachi, Pakistan; 5 Department of Global Health, Institute of Public Health and Nursing Research, University of Bremen, Bremen, Germany; 6 Heidelberg Institute of Global Health, University of Heidelberg, Heidelberg, Germany; 7 NLR International, Amsterdam, the Netherlands; 8 Erasmus MC, University Medical Center Rotterdam, Rotterdam, the Netherlands; 9 Enabel - Belgian Agency for International Cooperation, Niamey, Niger; 10 Care and Public Health Research Institute (CAPHRI)/Mental Health and Neuroscience Research Institute (MHeNs), Faculty of Health, Medicine and Life Sciences, Maastricht University, Maastricht, the Netherlands; London School of Hygiene and Tropical Medicine, UNITED KINGDOM OF GREAT BRITAIN AND NORTHERN IRELAND

## Abstract

Countries with low endemicity for leprosy face context-specific challenges in interrupting the transmission of *Mycobacterium (M.) leprae* to reach subsequent non-endemic status. This study explores the tensions and synergies that arise in resource-limited, low endemic settings when implementing and scaling-up single dose rifampicin (SDR) post-exposure prophylaxis (PEP) for leprosy control and elimination. We conducted 12 semi-structured in-depth interviews with key informants across six countries on the challenges and enablers in active case detection (ACD) and SDR-PEP implementation in low endemic countries. Key informants included medical practitioners, programme coordinators, researchers, policy makers, and experts, all with experience in PEP. Findings showed that lowering endemicity led to a fall in disease prioritization, lower levels of disease awareness within policy and healthcare practice, and the challenge of limited funding and resource accessibility. Advocating for leadership in leprosy control within national government and policy was central to long-term SDR-PEP implementation success, allowing for local alignment of policy and greater flexibility to adjust to changing disease patterns, resource availability and population health needs and priorities. Strengthening active coordination between stakeholders, both nationally and internationally, is vital for rifampicin procurement and supporting the integration of PEP into routine programmes. If the interruption of *M. leprae* transmission – and eventually leprosy disease elimination – is to be achieved, low endemic settings must also be considered in policy and practice. Our results provide key considerations for improving SDR-PEP implementation, specific to low endemic settings.

## Author summary

Despite global efforts, leprosy transmission remains static and high globally, with low-resourced, low endemic settings being disproportionately overlooked in policy and research as an opportunity to invest into effective leprosy control efforts. These settings face unique barriers to implementing post-exposure prophylaxis (PEP) with single dose rifampicin (SDR), a strategy proven to reduce transmission among contacts of leprosy patients. This study addresses this critical research gap by exploring the experiences, through in-depth interviews, of key stakeholders, including programme implementers, policymakers, and leprosy experts involved in active case detection (ACD) and SDR-PEP delivery across six low endemic countries. The study shows how diminishing leprosy endemicity leads to reduced funding, lower prioritization for leprosy control efforts by governments, and lack of awareness and expertise in the public and medical field, complicating the continuity of ACD and hindering the routine use of SDR-PEP for contacts of leprosy patients. Our findings reveal key insights into the structural, operational and financial tensions surrounding resource allocation and accessibility unique to low-endemic settings. In conclusion, a focus is needed on how to reposition leprosy control and SDR-PEP as a priority for funding and resources, particularly for high-risk and neglected populations, by ensuring governmental support and fostering national leadership in leprosy control. Our recommendations emphasise the importance of integration and cross-stakeholder coordination, both within and across countries, to secure resources, in particular rifampicin, to allow for routine implementation of SDR-PEP, thereby reducing transmission and contributing to the future elimination of this disease.

## Introduction

Leprosy is a communicable Neglected Tropical Disease (NTD) caused by infection from the bacilli *Mycobacterium (M.) leprae* or *M.lepromatosis* [1,2]. It is thought to be transmitted via droplet transmission from untreated patients with a high bacterial load and prolonged contact (over 20 hours/week) with a confirmed case [3]. Leprosy can result in permanent damage to the skin, nerves, limbs, eyes, and respiratory tract [2,4]. Left untreated, leprosy can lead to chronic disability, social exclusion, and marginalisation [4]. Leprosy remains endemic in over 120 countries globally and it is estimated that currently over 3–4 million people worldwide are disabled by the consequences of leprosy [5]. This highlights the importance of prevention, early diagnosis and effective treatment for leprosy [2,6].

Thanks to the introduction and free distribution of multidrug therapy (MDT) by the World Health Organisation (WHO) since 1982, leprosy is now a curable disease and patients using MDT are no longer contagious [7,8]. In addition, at the 1991 World Health Assembly, the target of ‘elimination of leprosy as a public health problem’ by 2000 (resolution 44.9) was set [9]. Elimination as a public health problem was defined as reaching a prevalence of less than 1 case per 10 000 population, which was achieved by most countries between 2000 and 2010 [10]. The achievement of this elimination target was mainly due to the reduced treatment duration following the introduction of MDT and the removal of cases from registers [11,12]. At the time, it was mistakenly believed that achieving the elimination target meant there would no longer be significant leprosy cases in those countries, a misconception that negatively affected funding and priority setting on public health agendas. Elimination of transmission (or interruption of transmission) has now been defined by the WHO Task Force as “reduction to zero of the incidence of infection caused by a specific pathogen in a defined geographical area, with minimal risk of reintroduction, as a result of deliberate efforts; continued actions to prevent re-establishment of transmission may be required” [13].

In the past two decades, the annual new case detection rate, a marker of active leprosy transmission, has remained static and high, with around 200 000 new cases detected annually at global level prior to the COVID-19 pandemic and 182 815 in 2023 [14,15]. Because leprosy continues to pose a health problem, and strategies aimed at early case detection and treatment are not sufficient to stop transmission and prevent infection, new strategies were needed, for example focused on leprosy prevention [16]. The COLEP study in Bangladesh (2008) showed that SDR-PEP reduces the risk of transmission by up to 57% among contacts of leprosy patients [17]. The LPEP study demonstrated that SDR-PEP is well-accepted and that it is feasible to implement it in routine leprosy control programmes in seven countries across three continents [18]. WHO’s 2018 ‘Guidelines for the Diagnosis, Treatment and Prevention of Leprosy’, the WHO 2021 technical guidance on ‘Contact tracing and Post-Exposure Prophylaxis’ and WHO’s ‘Roadmap for NTDs 2021-2030’ all endorse combining ACD with SDR-PEP for prevention [19–21].

Low endemic countries are referred to as areas that are reaching the interruption of transmission but have not yet achieved complete elimination of the disease [5,12]. Thanks to various factors, including improved global welfare and innovations in leprosy prevention, it is expected that in the upcoming years, more countries will become low endemic for leprosy.

There are various approaches to implement ACD and SDR-PEP administration, tailored to different settings and levels of endemicity. Approaches outlined by a recent study by ter Ellen et al. (2022) and in the WHO technical guidance (2023) are: the close contact approach (requiring consent of the index patient to disclose their disease status to close contacts), the blanket approach, and community-based skin camps [6,12]. The blanket approach administers SDR-PEP to an entire community, avoiding individual disclosure but requiring significant resources, making it suitable for hot-spots or remote areas. Community-based skin screening events, also called skin camps, screen for multiple skin diseases in large groups, reducing stigma around leprosy but needing substantial funding and logistical support. Each method provides unique benefits depending on setting and resource availability.

Most international studies on leprosy post-exposure prophylaxis (PEP) focus on high-endemic regions [17,18,22–25]. Yet, research in low-endemic settings is critical for tailoring policy and eventually achieving interruption of *M. leprae* transmission. Morocco’s success in interrupting leprosy transmission shows that a national screening and SDR-PEP approach for high-risk contacts can quickly reduce leprosy without extensive resources [26]. Our study aims to address implementation challenges of PEP in low-endemic contexts, where uptake of SDR-PEP is limited, by gathering insights from experts and stakeholders, and examining strategies that work effectively within such environments, aiming for sustainable leprosy control programs [6,27,28]. For this study, successful implementation is defined as; i) implementing ACD as a routine intervention across the country, ii) administrating SDR-PEP to all eligible contacts of index cases, and iii) achieving a reduction in national leprosy transmission and burden. This research will help inform future planning in order to strengthen and scale up ACD and PEP in low endemic settings.

## Methods

### Ethics statement

Ethical approval was obtained from the Global Health Ethical Review Committee at Maastricht University (registration number FHML/GH_2023.028; date of approval 23/05/2023). The study was conducted in accordance with the Research Data Management Code of Conduct by Maastricht University. Written informed voluntary consent was acquired prior to each interview, with information provided on the purpose of the study, selection criteria, data collection procedures, right to withdraw, and potential risks stated clearly. For the audio recording of the interviews, specific written and verbal consent were obtained before starting the discussion. All participants of the study had to be over 18. ID codes were used for pseudonymization. Confidentiality was kept for all stored data from individuals as per the General Data Protection Regulation (GDPR).

### Theoretical framework and study design

**Fig 1:**
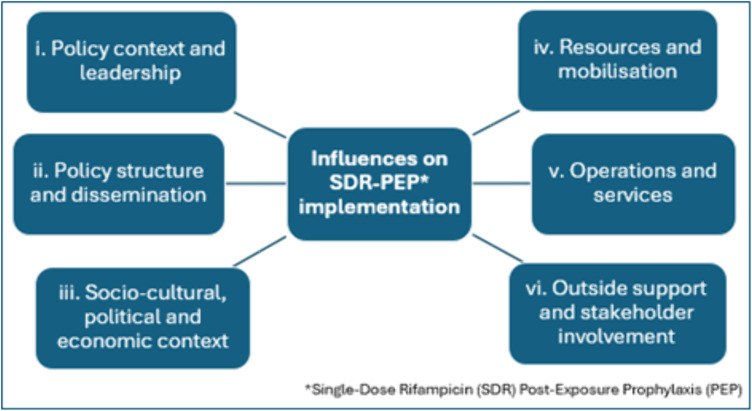
Themes included in the theoretical framework that influence the process of SDR-PEP across settings.

Using existing literature, we built a theoretical framework to explore the process of SDR-PEP implementation. We developed themes around the factors that contribute to SDR-PEP adoption, integration, functioning and success at national, regional and local levels [6,29–37]. An interview guide was then constructed based on these themes: (i) policy context and leadership; (ii) policy structure and dissemination; (iii) social, cultural, political and economic context; (iv) resources and mobilisation; (v) operations and services; and (vi) outside support and stakeholder involvement (see Fig 1).

Consequently, the themes identified in the theoretical framework, and reflected in the interview guide, allowed for a deeper understanding and insight into the interconnections between policies and the practical implementation of SDR-PEP. This was achieved using a qualitative study design to explore the perspectives and experiences of the various stakeholders through semi-structured, in-depth interviews. Participants were asked to explore their views on what makes a successful PEP programme and what has facilitated success.

### Study setting

**Fig 2:**
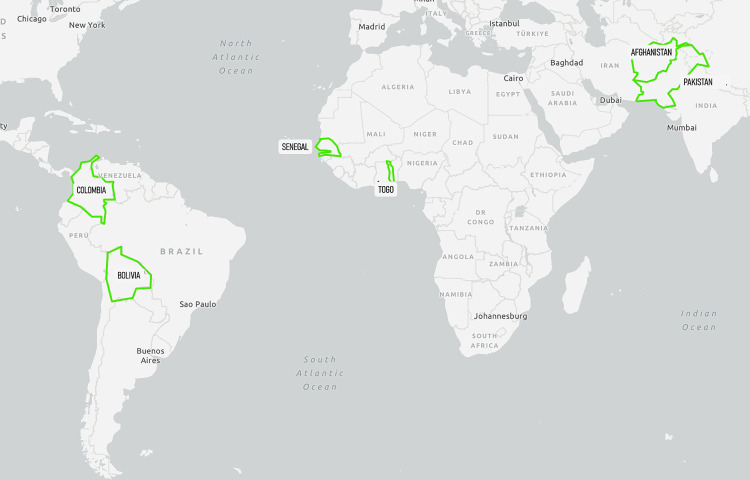
Visual representation of the countries selected for this study. Map designed using U.S. Geological Survey (USGS) [Accessed 09/09/2025] http://www.usgs.gov.

Data collection for this study took place online through the tele-conferencing programme Zoom from May 2023 to July 2023. Six countries were included in this study; Afghanistan, Bolivia, Colombia, Pakistan, Senegal, and Togo, covering the WHO Eastern Mediterranean Region (EMR), African Region (AFR), and Region of the Americas (AMR) (see Fig 2). Purposive sampling was used to select these countries based on active participation in leprosy control programmes or plans to implement SDR-PEP, geographical spread and low endemicity (based on WHO 2022 statistics) (see Table 1). Convenience sampling was used as all countries were in contact with the German Leprosy and Tuberculosis Relief Association (DAHW). The countries chosen are all varied in socio-cultural context, demography, geography, and political environment thereby adding diversity to the sample. This allows for better translatability of policy recommendations to a wider range of contexts at the end of the study.

**Table 1:** Number of new leprosy cases in 2022 according to the WHO for the countries selected [38].

WHO Region	Country Name	Number of new leprosy cases	Leprosy registered prevalence rate per 1 000 000 population
AFR	Togo	86	9.72
AFR	Senegal	161	10.68
AMR	Bolivia	38	4.91
AMR	Colombia	294	6.28
EMR	Afghanistan	7	0.22
EMR	Pakistan	259	1.39

Abbreviations: EMR = Eastern Mediterranean Region, AMR = Region of the Americas, AFR = African region.

### Sampling strategy and research population

12 key informants from the included countries were selected based on; i) their role in public health or the national leprosy control programme, ii) knowledge on SDR-PEP, iii) willingness to participate, and iv) availability and communicability [39]**.** The purposive selection of key informants was restricted to experts in the field that have experience with the current WHO Leprosy guidelines and implementation or planning of SDR-PEP and those who work at policy or project management level.

The 12 participants interviewed included experts working for various non-governmental organisations (NGOs), the Ministry of Health (MoH), the country’s National Leprosy Programme (NLP), in research, in policy making or in the medical field (see Table 2). Some participants worked in multiple capacities or for more than one organisation, denoted by the code names in bold in the table below. Due to availability issues, only one key informant was able to participate from Bolivia. A separate informal discussion with the DAHW representative of Bolivia took place to ensure the validity of the data collected.

**Table 2:** Affiliation of all study participants based on data collected during the interviews. Those working in multiple capacities or for more than one organisation are denoted in bold.

	Affiliation of expert				
Country	Medical Field	NGOs	Research	NLP	WHO policy
Afghanistan (n = 2)				A13	A4, **A13**
Bolivia (n = 1)		B6		**B6**	
Colombia (n = 2)	C11			**C11,** C12	
Pakistan (n = 3)	P3	P1, P2		**P1, P2**	**P3**
Senegal (n = 2)			S10	S9	
Togo (n = 2)		T7	T8	**T7**	
*Total in each field*	*(n = 2)*	*(n = 4)*	*(n = 2)*	*(n = 8)*	*(n = 3)*

### Data collection

Interviews lasted around 45 minutes and were conducted in English. The first author of this study led the interviews, with the assistance of an interpreter in four of the interviews (French and Spanish). The interviews were all audio-recoded and transcribed verbatim. Reflective observation notes were made during the interviews. One interview was only half recoded due to battery issues – the reflective observation notes serve as the record for the rest of this interview. A short summary of the key points raised were discussed with the interviewee on completion of the interview to check for accuracy.

### Data analysis

Data collection and analysis followed a cyclic pattern, involving the theoretical framework (see Fig 1), the design of the research instrument, participant recruitment, qualitative data collection, and the subsequent generation of inductive inferences to discover trends and guide thematic analysis [40]**.** Through incorporating data analysis in data collection, the interview guide was subsequently adapted and improved to take account of developing themes, such as the tensions surrounding integration of SDR-PEP into other NTD programmes, thus generating richer data as the process continued [40].

Each theme and sub-theme were coded and organized into overarching domains using the computer assisted qualitative data analysis software NVivo 12. After the first two interviews were coded, they were validated by two co-authors to determine whether the codes used were appropriate; resulting in the addition of a few supplementary codes. Horizontal analysis was performed by comparing the interviews with each other. The key themes were then described and used to conceptualize the findings and show how they either contribute to existing theory or develop new theory which could eventually lead to policy recommendations [40].

## Results

### Stages of ACD and SDR-PEP implementation

The table below shows the different stages of implementation of both ACD and SDR-PEP implementation across the various countries at the time of the interviews (June to July 2023) (see Table 3). The results are based on respondents’ answers (cross-checked with responses from all participants) and checked against any existing policy literature or guidelines to ensure there were no discrepancies. The X-marked boxes represent the stage which a country has reached at that time. All countries included in the study have achieved routine implementation of ACD in all or part of their country as the first step towards routine SDR-PEP implementation. Pakistan is currently the only country in this study to have reached routine implementation of both ACD and SDR-PEP.

**Table 3:** The stages of SDR-PEP implementation and stages of active contact tracing in the study’s countries. Results based on a combination of participant’s answers and research of policy literature. Data collected between June and July 2023.

	Afghanistan		Bolivia		Colombia		Pakistan		Senegal		Togo	
	SDR-PEP	ACD	SDR-PEP	ACD	SDR-PEP	ACD	SDR-PEP	ACD	SDR-PEP	ACD	SDR-PEP	ACD
**Research**			X		X						X	
**Implementation stage/ Implementation pilot**	X											
**Routine practice in some settings**		X		X					X			
**Routine practice across the entire country**						X	X	X		X		X

Abbreviations: SDR-PEP = Single Dose Rifampicin Post-Exposure Prophylaxis, ACD = Active Case Detection.

### Themes

The findings were based on themes from the theoretical framework and inductive analysis of data, and include: i) prioritizing leprosy, ii) integration of leprosy into other health programmes, iii) resources, iv) surveillance and monitoring systems v) local alignment of policy, vi) establishing focal points, and vii) fostering trust.

#### Prioritizing leprosy.

Participants explained that priority-setting for leprosy is globally guided by the WHO and ILEP through their published NTD Roadmap. However, the decision to implement the guidelines, and to what extent they are adopted ultimately lies with the respective country’s government and policy makers (B6). Due to its low endemic status, participants from all countries highlighted that policy makers often remain uninformed as to the consequences of leprosy and the resources needed by the healthcare system to effectively implement SDR-PEP. When there is a lack of awareness regarding leprosy’s ongoing public health impact, governments struggle to formulate an effective list of priorities. This, in turn, often results in inadequate resource allocation, impeding the scale-up of SDR-PEP implementation.

*‘[Policy makers] can prioritize whether leprosy will be included and how much of those resources can be designated for leprosy. Some of them haven’t even seen or heard of leprosy at all in their lives. (…) And of course, this adds up to the problem of putting leprosy in the public health agenda.’ (C11)*

In addition, participants highlighted that the low priority assigned to leprosy was attributed to its low national prevalence, despite the presence of disease hot-spots. It was asserted that with a low caseload, it is increasingly difficult to convince governments to consider leprosy as a priority and invest in preventative interventions such as SDR-PEP, particularly when other diseases are equally or more prevalent and destructive to population health overall (A4). Participants emphasized the pressing need to educate policy makers (e.g., Ministry of Health and the Ministry of Social Affairs) that a low case load does not mean that the disease does not hold significant health, economic and social consequences (S9).

Participants also expressed concern that without the ‘proper mainstreaming of leprosy’ in the public health agenda, healthcare workers lack a heightened awareness of the possibility of leprosy, facing the grave risk that it will be overlooked, and no effective treatment or control measures will be administered in sufficient time, even if SDR-PEP is fully operational (P1).

‘*There are fewer and fewer cases, so there are fewer and fewer health personnel who know about leprosy, who know how to recognize a person with leprosy cases. It’s getting more and more difficult, more challenging. Putting leprosy on the public health agenda, keeping that as a priority, and above all, generating capacity and knowledge of the disease by health personnel is what we need [for the success of SDR-PEP].’ (C11)*

In addition, participants discussed the risk of healthcare workers choosing to invest time, effort, and resources in other more ‘visible’ and ‘painful’ diseases, especially in the context of resource-tight settings.

*‘The issue is that most people think that leprosy has been eradicated and this is not a common disease. And then the other thing is, which is very common, that since this is in the early stages a very painless disease, people take it very lightly. This is the reason that people, including healthcare workers, say leprosy is not a disease which we must put on our top priority.’ (P2)*

Another challenge exacerbated by lowering endemicity, is the wider geographical spread of the remaining cases, with disease hot-spots remaining in remote, difficult to access locations. As described by participants from Afghanistan, Bolivia, and Pakistan, those living in these hot-spots are often from disadvantaged socio-economic backgrounds, hindering their access to medical care even when it may be available at a centralized location and free of charge (A4, B6). Investing in mobile health clinics and training in remote, hot-spot areas was one option proposed for diminishing access barriers for underserved groups to interrupt transmission in disease epicentres (C11).

#### Integration of leprosy into other health programmes.

WHO’s *Roadmap to End NTDs by 2030* recommends the integration of several NTD interventions onto a single NTD platform allowing for centralized planning and shared case-finding [20]. Participants from Colombia, Togo, and Pakistan argued that integrating ACD and SDR-PEP with other diseases provided benefits in sharing resources in a flexible and quick manner. For example, Togo integrated ACD for leprosy with Buruli ulcer and yaws which has improved programme efficiency through joint interventions (T7). This combined approach has also garnered greater governmental awareness and funding for SDR-PEP implementation compared to when the leprosy programme functioned alone (T7).

Integration of leprosy control into other high priority public health programmes, such as that of TB, also proved effective in overcoming the lack of direct funding for SDR-PEP implementation by the government (C11, A4). Consequently, adopting a resource-sharing approach has allowed programme coordinators to access a common public health fund for SDR-PEP interventions when needed.

Integrating NTD training, including the basics of leprosy identification, into general medical training was proposed to strengthen ACD and PEP administration. Participants emphasized that by doing so, all healthcare professionals would be more effective sentinels for leprosy and would be able to refer cases more quickly, assisting in early diagnosis and treatment. In this context, leprosy control in Pakistan has collaborated with primary eye care services, resulting in the creation of ‘leprosy ophthalmic technicians’. This has extended leprosy screening and care to a broader population, including access to remote areas through the pre-existing care structure of primary eye care (P2).

Furthermore, integrating leprosy awareness into broader NTD campaigns enabled countries such as Senegal to broaden the coverage and dissemination of leprosy information while simultaneously avoiding the potential for increased stigmatization that might result from having a separate campaign for leprosy.

*‘There have been mass campaigns for NTDs and other diseases, and it’s important that the preventive program against leprosy becomes an integral part of any national campaign. There have been youths in the villages who said, look, there are mass campaigns about various diseases, why on earth does leprosy have its own campaign, which of course makes it more susceptible to being stigmatized.’ (S9)*

On the other hand, some participants held the viewpoint that if leprosy was fully integrated into a shared monitoring structure with other NTDs it could hinder timely leprosy case reporting due to bureaucratic delays. In this case, participants advocated for a vertical system for leprosy case reporting, where cases are monitored separately, allowing SDR-PEP implementation efforts to target hot-spots, using limited resources efficiently (A13). These participants remained open to the idea of total integration only if clear policy from the WHO was provided and disseminated to public health surveillance and monitoring services.

In addition, a lack of clear policy from the WHO regarding rifampicin supply was also identified as a barrier when integrating leprosy control with other skin related NTDs that require a treatment regimen also including rifampicin, such as Buruli ulcer and TB. Participants expressed that exchanging medication (in this case rifampicin) between disease programmes, as a form of disease integration, could foster medication availability and thus PEP upscaling in leprosy control.

*‘I always complain to WHO saying that; you guys are promoting the use of single dose rifampicin to prevent transmission. Yet, you don’t provide rifampicin on a routine basis for leprosy. But at the same time, we have rifampicin for tuberculosis, for Buruli ulcer, but you are not allowed to use tuberculosis rifampicin to treat leprosy patients. We are not allowed to use Buruli ulcer rifampicin to treat leprosy. (…) I’m asking loudly to whoever would want to hear, the WHO or ILEP members, are we really promoting integration just on paper? And in practice does it happen? Nobody seems to provide a clear guideline on how.’ (T7)*

In summary, promoting integration of SDR-PEP into other public health programmes encourages resource sharing and ensures the long-term sustainability of the intervention. However, integration needs to be implemented effectively with clear unified aims.

#### Procurement of rifampicin.

As mentioned in the previous section, one of the key resources necessary for the full and effective functioning of SDR-PEP is the constant availability of sufficient MDT to treat leprosy patients as well as single-dose rifampicin for PEP. Many participants expressed concerns about the procurement of rifampicin, noting that the drug is not produced in sufficient quantity to ensure a constant and adequate supply for SDR-PEP, particularly in low endemic settings. This was attributed to administrative obstacles (rifampicin not being registered for leprosy prevention by the regulatory authorities), lack of political advocacy for drug procurement to international pharmaceutical companies, unfavourable demand for pharmaceutical companies to produce rifampicin in larger quantities for low endemic areas, concerns about rifampicin resistance, and competition with other disease programmes over the available supply of the drug, for example for rifampicin reserved for TB treatment, which is more endemic than leprosy.

*‘A big challenge is that we don’t have rifampicin on a routine basis, the rifampicin for SDR-PEP. Even though global partnership for leprosy is promoting the administration of single dose of rifampicin to prevent the transmission, and WHO is promoting it, but where are we getting the rifampicin?’ (T7)*

Participants emphasized the need to develop ‘high-quality affiliation’ with pharmaceutical companies to produce sufficient supplies of rifampicin if SDR-PEP is to be implemented as a routine intervention (A4). This is particularly important as rifampicin is used for both the prevention and treatment of the disease, so relying upon disease burden alone may not encourage sufficient production of the drug, especially in low endemic settings.

A potential solution for low endemic settings that struggle to procure rifampicin is to consider local production of the drug (A13, B6). This would provide greater control over its production and cost. Yet, most countries do not have the resources to produce rifampicin locally under franchise agreements, or the quality of the local rifampicin is unclear.

*‘Even if I can find rifampicin here in the country, I will not be allowed to purchase any medicine which does not have quality certificate. (…) If quality certificate is not available, as a WHO partner, I will not be allowed to purchase such medicine. So, I will need to have procurement from international market.’ (A13)*

Currently, the provision of rifampicin for SDR-PEP in most low endemic countries relies on charitable institutions. Participants expressed growing concern that this creates a dependence upon outside charitable assistance, which if lost, will result in SDR-PEP programme collapse. Some participants added that this dependency reduces pressure on governments to introduce their own nationally funded schemes.

In many of the countries in this study, where leprosy is present, TB is also endemic. The rifampicin used to treat TB, as well as leprosy, comes in combination tablets/capsules or in combined blister packs. However, for PEP, loose rifampicin is needed. This is further challenging SDR-PEP scale-up as rifampicin itself may be available on the market, but the type might not be suitable.

*‘It is very difficult to find plain rifampicin because it was a drug that was normally used in the treatment for tuberculosis, so it comes with several combinations. (…) Now we try to manage by placing orders from the pharmaceutical companies that we want to arrange this drug for specifically this use and then they will make the availability for that hopefully.’ (P2)*

Participants were deeply concerned about the ethical dilemma of carrying out contact screening without rifampicin being available when contacts were identified.

*‘Contact tracing and PEP should go hand in hand. Because if you go and trace contacts and then you don’t have anything to provide (…) is it really necessary to go for contact tracing? (…) We ultimately should have a procurement for rifampicin on a routinely basis. (…) Because if I have a leprosy case and then I have a contact and then you ask me, what next? I should have rifampicin. So they go hand in hand.’ (T7)*

Yet, one participant acknowledged that even if the drug is not currently available for widespread PEP use, unless screening and diagnosis takes place, the extent of the burden of the disease will not be recognized and there will be no pressure upon pharmaceutical companies to produce the drug at an affordable price and in an accessible way for low endemic countries (T7).

Regulatory authorities responsible for rifampicin access, along with advocacy efforts directed at pharmaceutical companies and governments, need to be at the forefront of SDR-PEP implementation strategies. Unless rifampicin is readily available, no prevention programme can be fully effective, however well planned.

#### Human resources and training.

There were two main challenges identified by participants in relation to human resources. The first was the shortage of leprosy specialists, such as dermatologists or expert NTD doctors, and the second was the shortage of female health workers. Furthermore, the low prevalence of leprosy in these countries has been seen as a reason not to invest additional funds in specialist training. This has resulted in leprosy being misdiagnosed or SDR-PEP being incorrectly prescribed due to the lack of familiarity and expertise with the disease, particularly in remote hot-spots (A4, S9, T8). All participants stressed the critical need to have adequate numbers of well-trained leprosy staff to diagnose and refer cases to specialists and implement SDR-PEP safely, with investment in training prioritizing difficult to reach, hyper-endemic areas of leprosy.

Another challenge is the shortage of female health workers, particularly at ground-level. Participants from Afghanistan, Pakistan, and Togo explained how male health professionals are forbidden from examining a female patient for contact screening as it would be considered culturally insensitive within the context of those communities.

*“Men are considered the head of the family and would not like you [as a male healthcare worker] to have access to their women. You are obliged to explain at length that his wife was in contact with leprosy. The difficulty is that you cannot have access directly to the woman without going through the man. The man has to give you the authorization to be able to question the woman. This causes an ethical problem.” (T8)*

Participants expressed the need to empower and train more female workers to ensure there is equal access to both genders when carrying out contact screening and SDR-PEP administration (A13, P3, T7 & T8).

To ensure the success of SDR-PEP implementation, investment into capacity building and training of more healthcare workers is essential, taking extra effort to address rural areas and the shortage of female health workers.

#### Surveillance and monitoring systems.

All participants agreed that monitoring systems are essential for improving SDR-PEP implementation in the long-term. Due to the low caseload, participants emphasized the need for effective mapping strategies to assist in finding and analysing disease hot-spots, particularly when forced with limited financial resources. One challenge identified was fragmentation among different monitoring systems within a country for ACD and disease prevalence, in addition to issues regarding the poor functioning of already present leprosy control data systems (C12, C11).

One participant pointed out that vital time is often lost between data collection (e.g., incidence levels) and analysis, which delays SDR-PEP administration to contacts, and consequently increases transmission rates (C12). In addition, the information stored in the database can also serve as a valuable tool for assessing progress and fine-tuning SDR-PEP implementation to provide more effective delivery of leprosy care (C11). This is of particular importance for low resource, low endemic settings due to funding and resource constraints.

Participants suggested that data related to ACD and PEP should be combined with, and collected at the same time and level as other national leprosy reports, to ensure all data are collated. Furthermore, participants from South America and West Africa believed that a unified national reporting system will further emphasize the increasingly routine nature of SDR-PEP to policy makers and the health system at large.

#### Local alignment of policy.

Currently the guidelines for leprosy control, including ACD and SDR-PEP implementation, are provided by the WHO in the NTD Roadmap [20]. These guidelines are primarily directed at high endemic settings but serve as overarching guidance for all settings irrespective transmission rates. Most participants agreed that the WHO guidelines for SDR-PEP were clear, however, they also emphasized that without the inclusion of local decision makers in adapting the policy to local settings, there is little hope that implementation will be successful. Participants reiterated that it is not just a question of involving the local decision makers in the implementation, but prior to that, involving them in discussions regarding the design of the SDR-PEP implementation plan, to best align with the local context (T7).

Participants noted that lack of implementation research in low endemic countries limits evidence-based decisions on choosing the best suited SDR-PEP implementation approach. Some countries (Bolivia, Colombia, Senegal, and Togo) have previously run or are currently undertaking pilot studies to identify challenges and enablers unique to implementing SDR-PEP as a routine intervention in low endemic settings. For example, pilot studies in Colombia revealed that case identification and SDR-PEP acceptance improved when door-to-door contact screening was carried out by a specialist dermatologist and a person with experience of leprosy (C12).

Currently, pilot studies are not officially part of the SDR-PEP implementation process. Yet, this pro-active approach allows for fine-tuning of national/regional guidelines and the adaption of ACD and SDR-PEP approaches to fit the local context, thereby improving implementation outcomes (T7).

#### Establishing focal points.

Participants emphasized that the success of SDR-PEP programme implementation was reliant on the capacity and performance at the national level, for example within the Ministry of Health, often through an established focal point with defined roles and responsibilities. For example, participants from Afghanistan highlighted the importance of establishing a national focal point to act as a bridge between WHO, who supports funding, medication availability, and resources (e.g., training) for the programme, and with the workforce, who are the backbone of the public health system and ground-level leprosy control. Additionally, as illustrated by a participant from Afghanistan, establishing an official administrative-level focal point increases unified dedication to implementing SDR-PEP, ensuring that someone within the government is responsible for the programme, thereby preventing the neglect of leprosy control due to its low endemicity. However, one challenge raised by participants was the difficulty of securing permanent funding for a key focal person responsible for SDR-PEP implementation and resource procurement within the government.

*‘They [the MoH] are not reluctant actually they are saying there are financial limitations, we cannot recruit some specific person, we do not have finances, we do not have resources to cover salary for such a person.’ (A13)*

Afghanistan has overcome this by assigning LEPCO, a leprosy NGO working within the country, as a temporary focal point for the advocacy of the programme’s needs. Although effective for the time being, concerns were expressed around the lack of influence a charitable NGO holds to advocate for the country’s needs within the international public health world. As this was not an official position, it was felt that leprosy control did not have a voice at international forums such as WHO meetings. This puts low resource settings at a greater disadvantage, as they are unable to advocate for funding and support for leprosy control irrespective of their level of endemicity (A4).

Another enabler that was put forward by participants is the value of setting up regional and local focal points alongside national ones. These focal points, such as regional programme coordinators or public health officers, will act as leprosy control strong-holds by taking ownership of any interventions implemented in their locality. Participants hoped that by being able to operate ACD and SDR-PEP successfully through a network of local focal points, a more robust foundation is laid for the long-term implementation of routine leprosy control and disease monitoring (B6, S9).

#### Fostering trust and addressing stigma.

The theme of building trust emerged inductively during the analysis of the data based on discussions around stigma, acceptance of treatment, and the socio-cultural and political context in general. Many of the participants referred to the ‘myths’, ‘beliefs’, and ‘rumours’ surrounding leprosy and the stigma attached to the disease and anyone suffering from it (B6, T8). These varying understandings of leprosy and its association with criminality or social disobedience were reported to adversely affect disclosure of contacts for ACD and the acceptance of preventative treatment (T8).

*“Leprosy is considered a curse in some communities, because he [the leprosy patient] has sinned. (…) Someone with leprosy is considered a thief. They say that is why you lose your fingers. There is this cultural connotation of leprosy that causes problems for contact tracing and accepting any treatment.” (T8)*

This was seen as a considerable challenge for SDR-PEP in some low endemic areas, particularly due to the lack of awareness of leprosy due to low national prevalence (S9, T7, T8). Participants suggested that adopting a hybrid approach to ACD, combining door-to-door visits and community skin screening events (for multiple skin-NTDs), would facilitate access for all groups of people regardless of the prevailing levels of stigma and awareness in the area (B6, P1).

*“Where there is more stigma, we have found it is better to do general skin camps because in every community, in every family, you have persons who have skin problems. And that is something which helps in overcoming stigma around leprosy as it is not singled out. (…) This is something which helps you to get a relationship with the communities and the trust of the community so that they say okay if you find a patient with any skin problem we will come to your centre for treatment” (P1)*

These community clinics also hold the advantage of providing an opportunity for providing health education and destigmatising information about leprosy and other NTDs.

## Discussion

This study widens the current scope of knowledge on how to effectively implement ACD and SDR-PEP in low-endemic settings so that interruption of leprosy transmission can be achieved. The findings emphasize that while there is significant potential for SDR-PEP to contribute to interruption of leprosy transmission in low-endemic settings, the tensions and synergies surrounding implementation processes must be addressed. There is a pressing need for updated and need-specific technical guidance, either by ILEP Technical Commission or the WHO, to support such settings in progressing through the stages of disease elimination.

### Political will and colliding priorities

This study showed how inadequate political will directly affects the implementation of new public health interventions into routine practice; particularly when the intervention focusses on control rather than treatment of a low endemic disease and the healthcare system already suffers from financial and resource constraints [12]. The result of weak political backing for leprosy control was that of reduced financial commitment by government stakeholders and poor accountability at policy level, thereby challenging long-term programme sustainability and the robustness of delivery systems to provide effective treatment and control. Participants reported that this led to vulnerable communities being overlooked, either unintentionally through lack of resources hindering access or due to disjointed leprosy surveillance services. This degree of government separation from its output, in this case due to lack of political will to facilitate leprosy control interventions, is described as a ‘hollow state’ by Milward & Provan (2000), where success relies on independent public services or external charitable organisations to deliver interventions with little top-down coordination or governance [41,42].

The availability of more resources (funding, human resources, medication, infrastructure, and technology) for SDR-PEP implementation was identified as a lever for change in leprosy control. This was seen in a few countries, with COVID-19 or political unrest resulting in a drop of a country’s economic reserve, causing fewer resources to be dedicated to health, particularly those assigned with low priorities [43]. To secure long-term resource allocation and the sustainability of ACD with SDR-PEP administration, prioritizing leprosy at the policy level requires a concerted effort to advocate for leprosy elimination and raise awareness of its significant social and economic consequences [44]. In addition, as ACD is expanded and improved, an increased population should become eligible for SDR-PEP [45,46]. This means any advocacy efforts must be carried out continuously, while adapting to the current needs of the affected population, until the point of elimination is achieved. Our research revealed that to promote leprosy as a priority in the political eye, advocacy interventions should be directed at both local governments and international stakeholders, particularly in relation to the procurement of rifampicin and funding. One opportunity identified was to advocate for leprosy through an already established spokesperson for TB, given the already high value placed on TB interventions within governments (TB is endemic in most countries in this study alongside leprosy). This further opens up a better placed position to advocate for a fair, integrated approach to procuring rifampicin (including the often-scarce syrup for children), used both for TB treatment and leprosy control, tackling the tensions around rifampicin being ‘reserved’ for one disease only. By pooling resources, both diseases can benefit from a larger and more efficient distribution system, leveraging existing infrastructures. Joint local production, strategic procurement partnerships with pharmaceutical companies and officially registering rifampicin for leprosy prevention at the national level would further streamline its availability for leprosy and TB control and reduce dependency donations and foreign suppliers.

### Strengthening leprosy control at the front-line

Suboptimal capacity for case detection and leprosy diagnosis were noted to challenge the operationalisation of SDR-PEP intervention plans, a finding consistent with existing literature [47,48]. Decentralising leprosy control and SDR-PEP decisions to local focal points were identified as ways to strengthen peripheral health systems, meet local needs, and improve access in remote areas.

This follows the ‘decentralisation and commonization model’, allowing primary care to provide services previously run at the tertiary and secondary levels and expand local control of resources, consequently improving equitable distribution of services [49–51]. The results also suggested that decentralisation could bring the added benefit of collective action and advocacy from smaller stakeholders, such as NGOs, civil society, and social groups (e.g., persons with experience of leprosy), that are traditionally excluded from national policy-making decisions. This can subsequently widen the scope of political participation at the local level and facilitate the shaping of service delivery outcomes to the needs of the setting [52]. Strong political commitment and stakeholder participation, along with stable resources and funding, are essential [53]. However, decentralization remains a longer-term goal due to the lack of widespread political support [6,54,55]. Decentralization would be efficient if a referral system is built, where specialists confirm presumptive leprosy cases or treat complex leprosy cases from primary health care.

When describing tasks related to ACD and prevention in leprosy efforts, it is beneficial for both advocacy and practical implementation to outline roles and responsibilities, similar to those in the “Roles and Responsibilities” section of the WHO’s technical guidance on ‘Contact Tracing and Post-Exposure Prophylaxis’ [21]. This approach helps establish clear functions across various levels of the health system, ensuring improved coordination and adaptability to local needs.

For task-shifting to be effective, primary healthcare staff must receive continuous training to recognize and manage rare diseases amid heavy workloads and competing health priorities typical of such settings. This is likely challenging in settings with low leprosy endemicity as well as with low health care resources. Well-established referral pathways are therefore crucial to support these workers. Innovations like tele-dermatology and mobile health solutions, including the WHO Skin NTD App, can offer valuable support by enhancing diagnostic capacity and treatment guidance for frontline healthcare workers [56].

### Strategies for surveillance and monitoring as a tool for success

Effective monitoring and data management systems are crucial for tracking ACD and SDR-PEP implementation, monitoring the activities and making adjustments based on local data [57]. The findings from this study show that there is a current lack of effective communication of leprosy data through referral systems from local to larger health care facilities, resulting in poor trend identification and response times for ACD and SDR-PEP interventions. While the free, online data collection system DHIS-2 exists in all endemic countries, merging epidemiological data and information on stock levels of MDT for both TB and leprosy, it was reported to not function effectively in remote locations, particularly where there is poor internet access, and it was felt to exclude data collection specifically for ACD and SDR-PEP eligibility as well as rifampicin stock levels. Ideally, stakeholders believed that monitoring and referral systems should be set up early in the implementation timeline to provide an evidence-base to inform operational parameters for further widespread implementation [55,58–60]. This would facilitate communications between the various focal points for identifying potential local improvements in SDR-PEP administration and the mobilisation of resources to respond to local pressures. Updating existing leprosy control data management systems for ACD and SDR-PEP interventions can draw on both the practical guide by Richardus et al., ‘Minimal essential data to document contact tracing and single dose rifampicin (SDR) for leprosy control in routine settings’ and the WHO’s guidance on contact tracing and post-exposure prophylaxis in the ‘Recording and Reporting’ chapter [21,61].

Another challenge identified was the migration of persons and its effect on ACD, calling for greater up-to-date coordination of ACD data within the larger surveillance system including in the post-elimination phase. Within this, recording whether contacts have been screened and were eligible for SDR-PEP was recommended [55,61]. To achieve this, investment into geospatial mapping of hot-spots, and the movement of contacts, was considered an essential next-step by participants if SDR-PEP efforts were to result in higher impact interventions and prompt case detection [62,63].

Reliable, ongoing data collection on leprosy control is also crucial for applying the WHO Technical Guidance on ‘interruption of transmission and elimination of leprosy disease’, which aids in prioritizing areas and tracking elimination progress [12]. The associated tools, the ‘Leprosy Elimination Monitoring Tool’ (LEMT) and the ‘Leprosy Programme and Transmission Assessment’ (LPTA), assist countries in classifying sub-national areas, assessing programme performance, and verifying progress. The guidance encourages critical actions to be taken in support of the development of a dossier for leprosy elimination verification. In addition, our study found that the potential for accelerating drug resistance to rifampicin through scaling-up SDR-PEP remains a concern for policy makers and leprosy control advocates, with the risk of jeopardising treatment for active TB and leprosy [18,64,65]. Current evidence suggests that SDR given to contacts of leprosy patients, in the absence of symptoms of active TB, poses a negligible risk of generating resistance in M. leprae and *M. tuberculosis* [65,66]. To mitigate risks, countries should invest in SDR-PEP administration training, which includes screening for ineligibility due to TB symptoms, and establish systems for monitoring antimicrobial resistance (AMR) [18,21,66]. Collaboration between leprosy and TB programmes, as also mentioned above linked to medication supply chain management, can further enhance both antimicrobial stewardship (AMS) and AMR monitoring, improving overall public health outcomes. As with all antimicrobial disease control, health policy makers who invest in AMR would facilitate evidence-based decisions that allow for pro-active change if resistance appears [67,68].

A challenge remains that good performance of such communication and monitoring systems relies on specific skills of data management and analysis of the persons responsible [20]. Programme coordinators or administrative staff should address this by integrating disease confirmation, monitoring systems at the local level, at least with other similar skin diseases, to allow for the transfer of skills and economies of scope [69]. This will also prepare them for future advances in healthcare digitalisation.

### Social dimensions of implementation success

This study found that stigma and fear of social exclusion accompanied with a diagnosis of leprosy remains a critical barrier to ACD and the uptake of SDR-PEP, especially in remote communities within limited exposure to health education and awareness efforts. Index patients are often more reluctant to disclose their diagnosis to social contacts or neighbours compared to household contacts [70,71]. In line with other studies, this was shown to improve with local efforts in health education and awareness tailored to fit the local context [67,72]. As community culture and engagement was identified a strong determinant of trust in public health interventions, leprosy control efforts should promote a dynamic and robust discourse around leprosy in the context of expressed beliefs, fears, and the socio-cultural link with health-seeking behaviour, including participation of persons affected by leprosy [18,67,73,74]. This will further facilitate the engagement of local leaders or religious persons as recommended in the WHO NTD Roadmap: ‘Engage with traditional healers’ to improve acceptability of SDR-PEP [20,75]. Practical strategies for improving uptake were also identified in the setting of stigma; for example by organising community screening activities, such as the blanket approach, community skin screening events (“skin camps”) and screening via door to door visits, removing the pressure on leprosy patients to disclose contacts and the fear of being ‘singled out’ [6,24,61,76,77].

### Strengths and limitations of the study

This was the first qualitative study to our knowledge that gained insight into challenges and enablers of SDR-PEP implementation in low endemic countries from the perspective of leprosy experts and programme coordinators. Topics extending to the socio-cultural and community dimensions (e.g., methods of introducing new interventions into existing services, systems, power structures, and communities) may be under-emphasized. As community and local engagement was found to be critical to successful implementation and programme sustainability: future research should use independent sampling frames to capture more varied views, particularly from community-based workers and persons affected by leprosy. Qualitative studies involving staff that support implementation at all levels (e.g. NGOs, healthcare staff, policy makers, community nurses, civil society) would strengthen the design of future implementation research and focus-points for ACD and SDR-PEP. To expand the geographical scope of this study, countries within three WHO regions were considered, improving the transferability of data to similar settings: future research could include all WHO regions to ensure equal representation. In addition, the primary source of participants was through contacts provided by the DAHW, which may introduce selection bias. This method could limit the diversity of perspectives, as only individuals associated with the DAHW were included. Consequently, the findings may not fully capture the views of all relevant actors, especially those in underrepresented or more remote regions. Future research could incorporate more varied recruitment methods to include a broader range of stakeholders, particularly those working in remote regions and those associated with different leprosy organisations.

### Recommendations

Based on the findings of this study on ACD and SDR-PEP implementation in low endemic settings, the following actionable policy recommendations are summarised below (see Table 4).

**Table 4:** Policy recommendations for implementing ACD and SDR-PEP in low endemic settings. Based on the results of this study.

Theme	Policy recommendation	Further actions
Rifampicin Access	**Ensure that quality-approved loose rifampicin can be accessed for SDR-PEP** as requested/ purchased by the Ministry of Health or programme coordinators.	1. **Provide support to the relevant regulatory authorities to officially register rifampicin** as the gold standard medication for leprosy treatment and prevention - to avoid tensions around rifampicin stocks being ‘reserved’ for a specific disease and not shared between programmes operating in the field. 2. **Promote local production of high-quality rifampicin** whenever possible. Encourage international actors/ civil society/ charity organisations to support capacity building for local production. 3. **Collaborate with, e.g., TB programmes** to foster greater tenders towards pharmaceutical companies and may ease supply chain issues such as the import process and stockkeeping.
Leadership and Prioritization	**Establish a dedicated leadership role for leprosy control in the Ministry of Health.**	1. **Secure funding** for the leadership role. 2. **Define role responsibilities** - the person can act as a **focal point and advocate for leprosy control within government.**
Integration	**Promote wider integration of SDR-PEP** as per the WHO recommendations (WHO, 2017b; WHO, 2020a). **Expand the scope of SDR-PEP integration into more areas of public health, beyond skin-related NTD programmes** to optimize resource utilisation and enhance programme sustainability.	Consider integration with: 1. **Established programmes that share public health interventions** (e.g., contact tracing, awareness campaigns including those for other stigmatized diseases) 2. **Similar disease pathogens and treatment** (e.g., TB) – improving multi-disease use of rifampicin
Feasibility and Acceptability	**Conduct more studies to ascertain the feasibility and acceptability of SDR-PEP (or other future prophylactic medication regimes for leprosy) in low endemic settings.**	1. **Encourage local/regional/national adaption of guidelines** to align to county’s context and needs of the patient community. 2. **Include studies on the local belief system around leprosy and engage religious or community leaders, persons affected by leprosy for awareness efforts where appropriate.**
Case detection capacity	**Focus investment into ACD through adopting hybrid approaches**, such as combining skin camps, door to door visits, and community awareness campaigns to promote early detection and ensure maximum coverage.	1. **Promote passive case detection** through community awareness programmes. 2. **Consider and move to integrate new mobile healthcare detection applications** for remote assessment of remaining or hard-to-reach cases. 3. **Strengthen people’s organisations** to reduce stigma and discrimination and improve case disclosure.
Prevention and Monitoring	**Establish a dedicated surveillance and monitoring system for leprosy ACD data. Consider the benefits of implementing a separate monitoring system for leprosy ACD data to ensure data is interpreted and acted upon promptly**, even if leprosy is integrated into another public health programme.	1. **Provide adequate funding for the provision and maintenance of a surveillance and monitoring system**. 2. **Include if contacts found during ACD are eligible for SDR-PEP and if they have accepted the treatment.** i. **Record the doses of SDR given to ensure repeat doses are avoided.** 3. **Promote effective mapping strategies** to assist in finding disease hot-spots, e.g., geo-mapping so that SDR-PEP can be implemented in a resource-efficient way.
Capacity Building	**Invest in capacity building and healthcare worker training for ACD and SDR-PEP administration, with particular focus on training female healthcare workers to promote gender equality and equal access to treatment.**	1. **Integrate training into routine care or together with other health programmes** (e.g. NTDs) to reinforce referral and disease confirmation at the primary level. 2. **Use mobile teams** to reach transmission hot spots, combining with other health services required in the area. 3. **Build a referral system that is accessible for remote health centres** to access specialised advice, potentially with the support of WHO-recommended mobile health applications.

## Conclusion

Our study provides insights and recommendations to improve ACD and SDR-PEP implementation in low-endemic and low-resource settings. As leprosy transmission is expected to further decrease globally, more countries with previously high-endemic status will face the challenges associated with diminishing endemicity. These challenges include the increased geographical remoteness of leprosy patients; lack of awareness and further dwindling leprosy expertise in the public, amongst healthcare staff and policymakers; reduced political and financial support for ACD and preventative interventions such as PEP; and an increased need to justify the operational and administrative costs of SDR-PEP implementation. A focus is needed on how to reposition leprosy control and SDR-PEP as a priority for funding and resources, particularly for high-risk and neglected populations, considering any competing interests such as rifampicin reserved for TB treatment. Organisational challenges and lack of governmental support for leprosy could be overcome by adopting an integrated approach best suited to the strengths of that country’s health system as also advised by the WHO; communal funding for NTD interventions, combined training of health professionals, integrated awareness campaigns and resource-sharing, with focus on the multi-disease use of rifampicin.
